# Hyperuricemia increases the risk of acute kidney injury: a systematic review and meta-analysis

**DOI:** 10.1186/s12882-016-0433-1

**Published:** 2017-01-17

**Authors:** Xialian Xu, Jiachang Hu, Nana Song, Rongyi Chen, Ting Zhang, Xiaoqiang Ding

**Affiliations:** 1 Department of Nephrology, Zhongshan Hospital, Fudan University, No.180 Fenglin Road, Shanghai, 200032 People’s Republic of China; 2 Shanghai Institute of Kidney Disease and Dialysis, No.180 Fenglin Road, Shanghai, 200032 People’s Republic of China; 3 Shanghai Key Laboratory of Kidney Disease and Blood Purification, No.180 Fenglin Road, Shanghai, 200032 People’s Republic of China

**Keywords:** Acute kidney injury, Hyperuricemia, Uric acid, Meta-analysis

## Abstract

**Background:**

Mounting evidence indicated that the elevated serum uric acid level was associated with an increased risk of acute kidney injury (AKI). Our goal was to systematically evaluate the correlation of serum uric acid (SUA) level and incidence of AKI by longitudinal cohort studies.

**Methods:**

We searched electronic databases and the reference lists of relevant articles. 18 cohort studies with 75,200 patients were analyzed in this random-effect meta-analysis. Hyperuricemia was defined as SUA levels greater than 360-420 μmol/L (6–7 mg/dl), which was various according to different studies. Data including serum uric acid, serum creatinine, and incidence of AKI and hospital mortality were summarized using random-effects meta-analysis.

**Results:**

The hyperuricemia group significantly exerted a higher risk of AKI compared to the controls (odds ratio OR 2.24, 95% CI 1.76-2.86, *p* < 0.01). Furthermore, there is less difference of the pooled rate of AKI after cardiac surgery between hyperuricemia and control group (34.3% vs 29.7%, OR 1.24, 95% CI 0.96-1.60, *p* = 0.10), while the rates after PCI were much higher in hyperuricemia group than that in control group (16.0% vs 5.3%, OR 3.24, 95% CI 1.93-5.45, *p* < 0.01). In addition, there were significant differences in baseline renal function at admission between hyperuricemia and control groups in most of the included studies. The relationship between hyperuricemia and hospital mortality was not significant. The pooled pre-operative SUA levels were higher in AKI group than that in the non-AKI group.

**Conclusions:**

Elevated SUA level showed an increased risk for AKI in patients and measurements of SUA may help identify risks for AKI in these patients.

## Background

Acute kidney injury (AKI) occurs commonly after cardiovascular surgery, in patients with sepsis, and after the administration of various nephrotoxins including contrast agents. The incidence of AKI has a significant effect on the outcomes. Prevention before any procedure is essential because no measures have been proven to effectively treat AKI. Therefore, if high-risk patients could be screened earlier, the clinician still would have opportunities to prevent AKI and further improve outcomes [1, 2].

Uric acid is an end-product of purine degradation and is excreted via kidney. Many epidemiologic studies have suggested that hyperuricemia is associated with hypertension, cardiovascular diseases, diabetes mellitus and the progression of chronic kidney disease [3–5]. In addition, it is found that hyperuricemia is associated with acute kidney injury (AKI) in various statuses [6–9]. This meta-analysis was conducted to estimate whether hyperuricemia is an independent risk factor for incidence and prognosis of AKI. This effort hoped to raise awareness of the importance of hyperuricemia in the developing AKI.

## Methods

### Search strategy and data sources

We performed a computerized search to identify relevant published original studies (1985 to May 2016). Pubmed, Web of Science, Cochrwane Library, OVID and EMBASE databases were searched using medical subject headings (MeSH) or keywords. These words were “acute kidney failure, acute kidney injury, acute kidney dysfunction, acute kidney insufficiency, acute tubular necrosis, acute renal failure, acute renal injury, acute renal dysfunction, or acute renal insufficiency” and “hyperuricemia, or uric acid”. This search was not limited to English language or publication type. We followed a prespecified protocol but this was not registered.

### Selection criteria

An initial eligibility screen of all retrieved titles and abstracts was performed, and only studies reporting the relationship between serum uric acid (SUA) and AKI were selected for further review. The following included criteria were used for final selection: (1) studies reporting the incidence of AKI and pre-operative SUA Levels, (2) studies using clear definition of AKI, and hyperuricemia, (3) studies providing detailed information about the incidence of AKI, and/or hospital mortality. We restricted our search to clinical studies performed in adult populations. Studies without clear grouping or animal experimental studies were excluded.

### Data extraction and quality assessment

Two reviewers (X.X.L and H.J.C) examined the studies independently, and disagreement was resolved by discussion. Data extraction included country of origin, year of publication, study period, study design, inclusion criteria, definition of hyperuricemia or grouping according to SUA, conclusions and patient characteristics (age and sex). Hyperuricemia was defined as SUA levels greater than 360-420 μmol/L (6–7 mg/dl), which was various according to different studies. The primary outcomes were odds ratio (OR) of SUA to predict incidence of AKI. The definition of AKI in all these included studied used the AKI network criteria [10] with minor modification and defined as an increase ≥0.3 mg/dL in the serum creatintine level within 48 h in the hospital or ICU (Table 1). The second outcomes included SUA levels in AKI and No-AKI group and hospital mortality in hyperuricemia and control group. The study selection, data extraction and reporting of results were all based on the Preferred Reporting Items for Systematic reviews and Meta-Analyses checklist [11]. The quality of the cohort studies was assessed independently by pairs of two authors, using the Newcastle-Ottawa scale (NOS) [12], which allocates a maximum of 9 points for quality of the selection, comparability, and outcome of study populations. Study quality scores were defined as poor (0–3), fair (4–6), or good (7–9).

**Table 1:** Characteristics of studies included in the meta-analysis

Authors (year)	Study period	Country	Study design	Sample size	Mean age (y)	Percentage of Male (%)	Inclusion criteria	Definition of hyperuricemia or grouping according to SUA	Definition of AKI	Mean baseline eGFR in HUA group (ml/min/1.73 m2)	Conclusions
Shacham, et al. (2016) [48]	2008–2015	Israel	Retrospective cohort	1372	62 ± 12	85	Acute STEMI patients requiring PCI	<4.7 mg/dl, 4.8–5.6 mg/dl, 5.7–6.6 mg/dl, >6.7 mg/dl	A rise in sCr >0.3 mg/d above the admission sCr within 48 h	79 ± 19, 75 ± 17, 70 ± 11, 63 ± 20 for 4 groups respectively	Elevated UA levels are an independent predictor of AKI
Cheungpasitporn, et al. (2016) [49]	2011–2013	USA	Retrospective cohort	1435	62 ± 16	60.3	All hospitalized adult patients without ESRD and AKI at presentation and trauma	<3.4 mg/dl, 3.4–4.5 mg/dl, 4.5–5.8 mg/dl, 5.8–7.6 mg/dl, 7.6–9.4 mg/dl, >9 mg/dl	An increase in sCr ≥0.3 mg/dL within 48 h or ≥1.5 times baseline within 7 days after admission date	89.5 ± 20.6, 88.1 ± 21.9, 79.3 ± 24.5, 71.7 ± 24.8, 58.6 ± 22.3, 53.2 ± 21.8 for 6 groups respectively	Elevated admission SUA was associated with an increased risk for in-hospital AKI
Otomo, et al. (2015) [6]	1981–2011	Japan	Retrospective cohort	59,219	58.6 ± 17.9	48.4	All hospitalized patients	The first stratum: SUA ≤2.0 mg/dL; the 12th stratum: SUA >7.0 mg/dL, with SUA levels in each succeeding stratum increasing by increments of 0.5 mg/dL	An increase ≥0.3 mg/dL in the sCr level within 48 h; or ≥1.5 times baseline within the prior 7 days; or urine volume of 0.5 mL/kg/h within 6 h	102 ± 50, 99 ± 44, 96 ± 45, 93 ± 38, 88 ± 31, 86 ± 34, 81 ± 28, 79 ± 29, 76 ± 28, 73 ± 28, 70 ± 27, 59 ± 34 for 6 groups respectively	SUA level could be an independent risk factor for AKI development in hospitalized patients
Liang, et al. (2015) [50]	2009–2014	China	Prospective cohort	59	37.3 ± 10.6	NR	Severe burn	NR	An absolute anincrease in sCr > 0.3 mg/dl from baseline within 48 h after injury	NR	Elevated SUA after injury due to hypoxia is closely correlated with early AKI after severe burns
Lee, et al. (2015) [7]	2006–2011	Korea	Retrospective cohort	2,185	63.6 ± 9.1	74.7	All patients undergoing CABG	NR	An increase in sCr of ≥0.3 mg/dL or ≥150% from baseline within the first 48 h after operation	NR	Preoperatively Elevated SUA was significantly associated with AKI and improved the ability to predict the development of AKI in patients undergoing CABG
Lazzeri, et al. (2015) [51]	2006–2013	Italy	Prospective cohort	329	77.2 ± 10.0	53.8	STEMI patients submitted to primary PCI	SUA ≤ 5.9 mg/dl, 6.0–7.4 mg/dl, >7.4 mg/dl	An absolute increase in sCr level of 0.3 mg/dl or more, or a relative increase in sCr level of 50% or more during the ICCU stay	42.8 ± 14.3, 42.5 ± 13.4, 40.8 ± 12.2 for 3 groups respectively	Uric acid helps in identifying a subset of patients at a higher risk of AKI and 1-year mortality.
Gaipov, et al. (2015) [52]	2011–2012	Turkey	Prospective cohort	60	56.7 ± 16.4	70.0	Patients undergoing cardiac surgery	NR	An increase in sCr by 0.3 mg/dL within 48 h or increase in sCr to 1.5 times baseline	NR	Uric acid seems to predict the progression of AKI and RRT requirement in patients underwent cardiac surgery better than NGAL
Barbieri, et al. (2015) [8]	2007–2011	Italy	Retrospective cohort	1,950	72.1 ± 8.7	NR	Patients undergoing coronary angiography and /or angioplasty with GFR ≤ 89 ml/min	SUA ≤ 5.5 mg/dL; 5.6–7.0 mg/dL; ≥7.0 mg/dL	An absolute ≥0.5 mg/dl or a relative ≥25% increase in the sCr level at 24 or 48 h after the procedure	NR	Elevated SUA level is independently associated with an increased risk of CIN
Guo, et al. (2015) [53]	2010–2013	China	Prospective cohort	1772	64.43 ± 11.35	76.5	Patients who underwent PCI	SUA > 7 mg/dL (417 μmol/L) in males and >6 mg/dL (357 μmol/L) in females.	an increase in sCr of >0.5 mg/dL from the baseline within 48–72 h of contrast exposure	71.08 ± 24.70	Hyperuricemia is associated with a risk of CI-AKI. Long-term mortality after PCI was higher in those with hyperuricemia than with normouricemia after adjusting.
Joung, et al. (2014) [54]	2011–2012	Korea	Retrospective cohort	1,094	63.0	62.2	Patients undergoing cardiovascular surgery	SUA > 6.5 mg/dL (preoperative) (6.0 mg/dL in women and 7.0 mg/dL in men)	An increase ≥0.3 mg/dL in the sCr level or ≥1.5 times baseline within 48 h	NR	Preoperative elevated serum uric acid is an independent risk factor for AKI in patients undergoing cardiovascular surgery.
Xu, et al. (2014) [55]	2005–2011	China	Retrospective cohort	936	65.2 ± 4.2	54.3	Old patients (≥60 years) undergoing CPB	SUA ≤ 384.65; 384.66–476.99; ≥477.00 μmol/L (males) SUA ≤ 354.00; 354.01–437.96; ≥437.97 μmol/L (females)	An increase in sCr ≥150% from baseline within the first 7 days after operation	73.8 ± 17.2, 69.3 ± 14.2, 61.5 ± 15.8 for 3 groups respectively	Pre-operative elevated uric acid is an independent risk factor of AKI after cardiac surgery in elderly patients
Liu, et al. (2013) [56]	2010–2011	China	Prospective cohort	788	62.8 ± 11.3	78.6	Patients undergoing PCI	SUA >7 mg/dL in males and >6 mg/dL in females	An increase in sCr of ≥ 0.5 mg/dL above the baseline value within 48–72 h after PCI	*Creatinine Clearance: 65 ± 24 ml/min	Hyperuricemia was significantly associated with the risk of CI-AKI in patients with relatively normal serum creatinine after PCI
Lapsia, et al. (2012) [57]	2004–2008	USA	Retrospective cohort	190	63.9 ± 0.9	62.1	Patients undergoing cardiovascular surgery	SUA ≥7 mg/dL	An absolute increase in sCr of ≥ 0.3 mg/dL from baseline within 48 h after surgery	47.6 ± 1.8	Preoperative SUA was associated with increased incidence and risk for AKI
Ejaz, et al. (2012) [58]	NR	USA	Prospective cohort	100	61.4 ± 1.4	60	Patients undergoing cardiac surgery with eGFR > 30 ml/min/1.73 m^2^	SUA < 4.53 mg/dL, 4.53–5.77 mg/dL, > 5.77 mg/dL	An absolute increase in sCr ≥ 0.3 mg/dL from baseline within 48 h after surgery	NR	Post-operative SUA is associated with an increased risk for AKI and compares well to conventional markers of AKI
Park, et al. (2011) [59]	2006–2009	Korea	Retrospective cohort	1,247	64.3 ± 11.9	62.3	Patients undergoing PCI	SUA ≥7.0 mg/dl for males and ≥ 6.5 mg/dl for females.	An increase in sCr of ≥0.5 mg/dl or ≥50% over baseline within 7 days of PCI	NR	Hyperuricemia is independently associated with an increased risk of in-hospital mortality and AKI in patients treated with PCI
Kim, et al. (2011) [60]	2007–2008	Korea	Retrospective cohort	247	46.1 ± 13.7	52	Acute PQ intoxication	SUA ≥7.3 mg/dL in men or ≥5.3 mg/dL in women	An increase in sCr of ≥0.3 mg/dL or ≥150% from baseline within 48 h after admission	NR	Baseline serum uric acid level might be a good clinical marker for patients at risk of mortality and AKI after acute PQ intoxication
Ben-Dov, I. Z., et al. (2011) [61]	1976–1979	Israel	Retrospective cohort	2449	58.8	50 ± 6	Patients in Lipid Research Clinic cohort	>6.5 mg/dL in men and >5.3 mg/dL in women	NR	93 ± 18 in men and women	SUA was found to be a strong predictor of acute renal failure
Toprak et al. (2006) [62]	2004–2005	Turkey	Prospective cohort	266	58.9 ± 7.4	61%	Nonemergency diagnostic coronary angiography with Scr > 1.2 mg/dl	>7 mg/dl in men and 6.5 mg/dl in women.	An increase of ≥25% in sCr over baseline within 48 h of coronary angiography	55.26 ± 13.7	Patients with hyperuricemia are at risk of developing CIN.

*Abbreviations*: *SUA* serum uric acid, *sCr* serum creatintine, *AKI* acute kidney injury, *CABG* Coronary Artery Bypass Grafting, *STEMI* ST-elevation myocardial infarction, *PCI* percutaneous coronary intervention, *NGAL* neutrophil gelatinase-associated lipocalin, *GFR* glomerular filtration rate, *eGFR* estimated glomerular filtration rate, *CIN* contrast-induced nephropathy, *CI-AKI* contrast-induced acute kidney injury, *PQ* paraquat, *NR* not reported

### Data synthesis and statistical analysis

Review Manager (RevMan, Cochrane Collaboration, version 5.3) and Comprehensive Meta-Analysis (CMA version 2.0, Biostat) were used to perform the meta-analysis. Pooled estimates were obtained for incidence of AKI and hospital mortality, which were reported using random-effects meta-analysis based on the methods of DerSimonian and Laird [13]. Meta-analyses were performed using OR for dichotomous outcomes. All confidence intervals (CI) were reported at 95%. *P*-value statistical significance was measured at 0.05. Heterogeneity across trials was evaluated with using the*I* ^2^ index and the Q test *p* value. A *p* value of less than 0.05 and an*I* ^2^ index of more than 25% indicated the presence of interstudy heterogeneity [14]. Publication bias was assessed by constructing a funnel plot and Egger’s regression test.

## Results

### Study selection

**Fig. 1:**
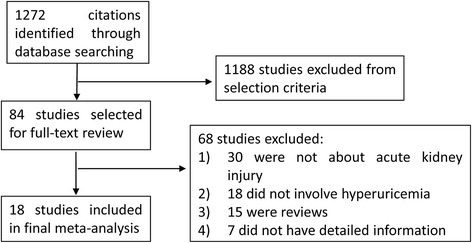
Flow chart of literature search and study selection

The article selection process is outlined in Fig. 1. The electronic database searches identified 1272 citations. After removal of duplicates and preliminary screening, 84 articles were selected for full-text review for their relevance to this study and 18 studies were included in this systematic review. At the full-text review stage, 30 articles were not about AKI, 18 did not involve hyperuricemia and 15 were review. Seven studies were excluded from the primary meta-analysis as they did not report the detailed information, and the corresponding authors were unable to provide the requisite data. Agreement between investigators at the full-text review stage was excellent as indicated by a κ of 0.8.

### Study description and quality assessment

A detailed description of the included studies is provided in Table 1. The included studies were published between 2006 and 2016, and were carried out in a wide range of countries. The total number of patients included in the primary meta-analysis was 75,200 with a median (interquartile range) of 559 (122–1774) patients per study. The detailed information of age and gender was also listed in Table 1. Overall study quality was good with a mean NOS score of 7.5 out of a possible 9 (range, 7–9) and with 11 studies (91.7%) receiving a NOS greater than or equal to 7 (Table 2).

**Table 2:** Quality of the studies utilizing the Newcastle-Ottawa quality assessment scale (Cohort studies)

Reference (Year)	Selection				Comparability	Outcome			Total score
	Representativeness of exposed cohort	Selection of the non-exposed cohort	Ascertainment of exposure	Demonstration that outcome was not present at start of study	Comparability of cohorts onthe basis of the design or analysis	Assessment of outcome	Follow up long enough	Adequacy of follow up of cohorts	
Shacham, et al. (2016)	☆	☆	☆	☆	☆☆	☆	☆	☆	9
Cheungpasitporn, et al. (2016)	☆	☆	☆	☆	☆☆	☆	☆	☆	9
Otomo, et al. (2015) [6]	☆	☆	☆	☆	☆☆	☆	☆	☆	9
Liang, et al. (2015)	☆	☆	-	☆	☆	☆	☆	-	6
Lee, et al. (2015) [7]	☆	☆	☆	☆	☆☆	☆	☆	☆	9
Lazzeri, et al. (2015)	☆	☆	☆	☆	☆	-	☆	-	6
Gaipov, et al. (2015)	☆	☆	☆	☆	☆	-	☆	-	6
Barbieri, et al. (2015) [8]	☆	☆	☆	☆	☆	☆	☆	-	7
Guo, et al. (2015)	☆	☆	☆	☆	☆☆	☆	☆	☆	9
Joung, et al. (2014)	☆	☆	-	☆	☆	☆	☆	-	6
Xu, et al. (2014)	☆	☆	☆	☆	☆☆	☆	☆	☆	9
Liu, et al. (2013)	☆	☆	☆	☆	☆☆	☆	☆	☆	9
Lapsia, et al. (2012)	☆	☆	-	☆	☆	☆	☆	-	6
Ejaz, etal (2012) [43]	☆	☆	☆	☆	☆	☆	☆	-	7
Park, et al. (2011)	☆	☆	-	☆	☆	☆	☆	-	6
Kim, et al. (2011)	☆	☆	☆	☆	☆☆	☆	☆	☆	9
Ben-Dov, I. Z., et al. (2011)	☆	☆	--	☆	☆	☆	☆	-	6
Toprakm, et al. (2006)	☆	☆	☆	☆	☆☆	☆	☆	☆	8

### Effects of SUA on the incidence of AKI

**Fig. 2:**
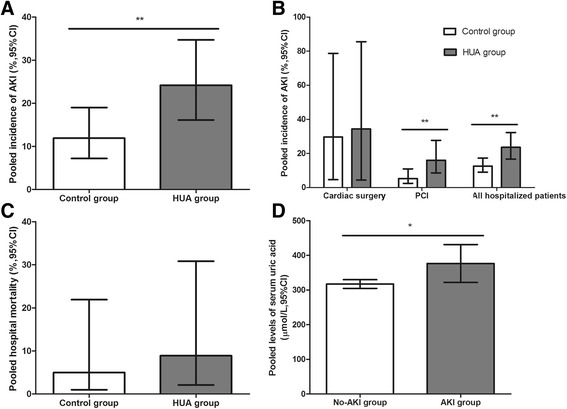
Hyperuricemia and acute kidney injury. **a** The pooled rates of AKI incidence in control and hyperuricemia (HUA) group; (**b**) Subgroup analysis in all hospitalized patients and patients with cardiac surgery and PCI; (**c**) The pooled hospital mortality in control and HUA group; (**d**) The pooled levels of SUA in No-AKI and AKI group. *p < 0.05, **p < 0.01

**Fig. 3:**
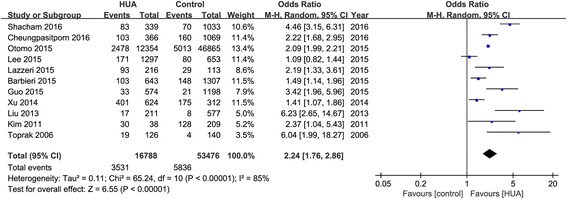
Effects of hyperuricemia on incidence of acute kidney injury

**Fig. 4:**
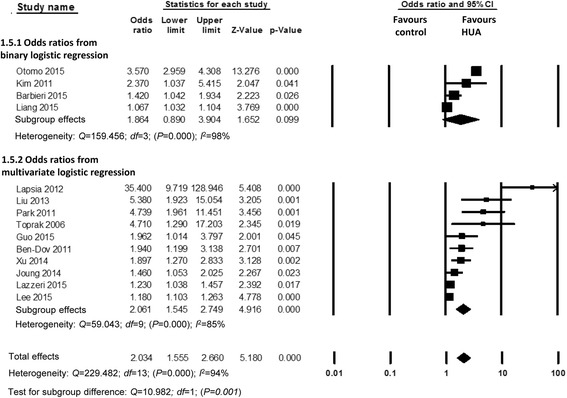
Pooled odds ratios of serum uric acid to predict acute kidney injury

Eleven observational studies with 70,264 patients reported the incidence of AKI. The pooled rates of AKI incidence in hyperuricemia group and control group were 24.2% (95% CI, 16.1-34.7%) and 11.9% (95% CI, 7.2-19.0%) respectively (OR 2.24, 95% CI 1.76-2.86, *p* < 0.00001) (Figs. 2a and 3). Four studies reported ORs of SUA to predict AKI by binary logistic regression and ten studies reported ORs by multiple logistic regression, and the pooled ORs were 1.864 (95% CI 0.890-3.904, *p* = 0.000) and 2.061 (95% CI 1.545-2.749, *p* = 0.000) respectively (Fig. 4).

**Fig. 5:**
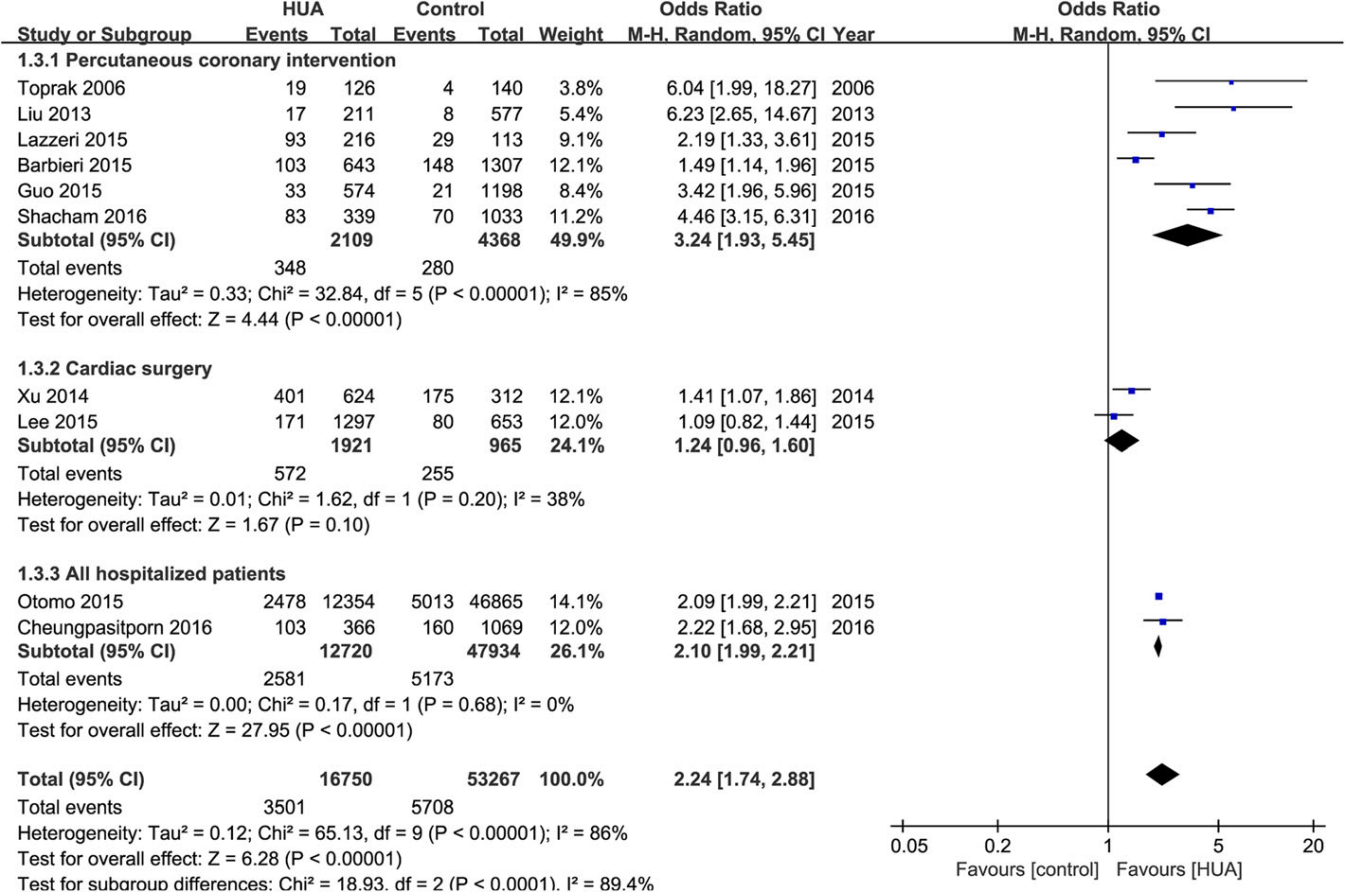
Effects of hyperuricemia on incidence of acute kidney injury in all and subgroup analysis

**Fig. 6:**
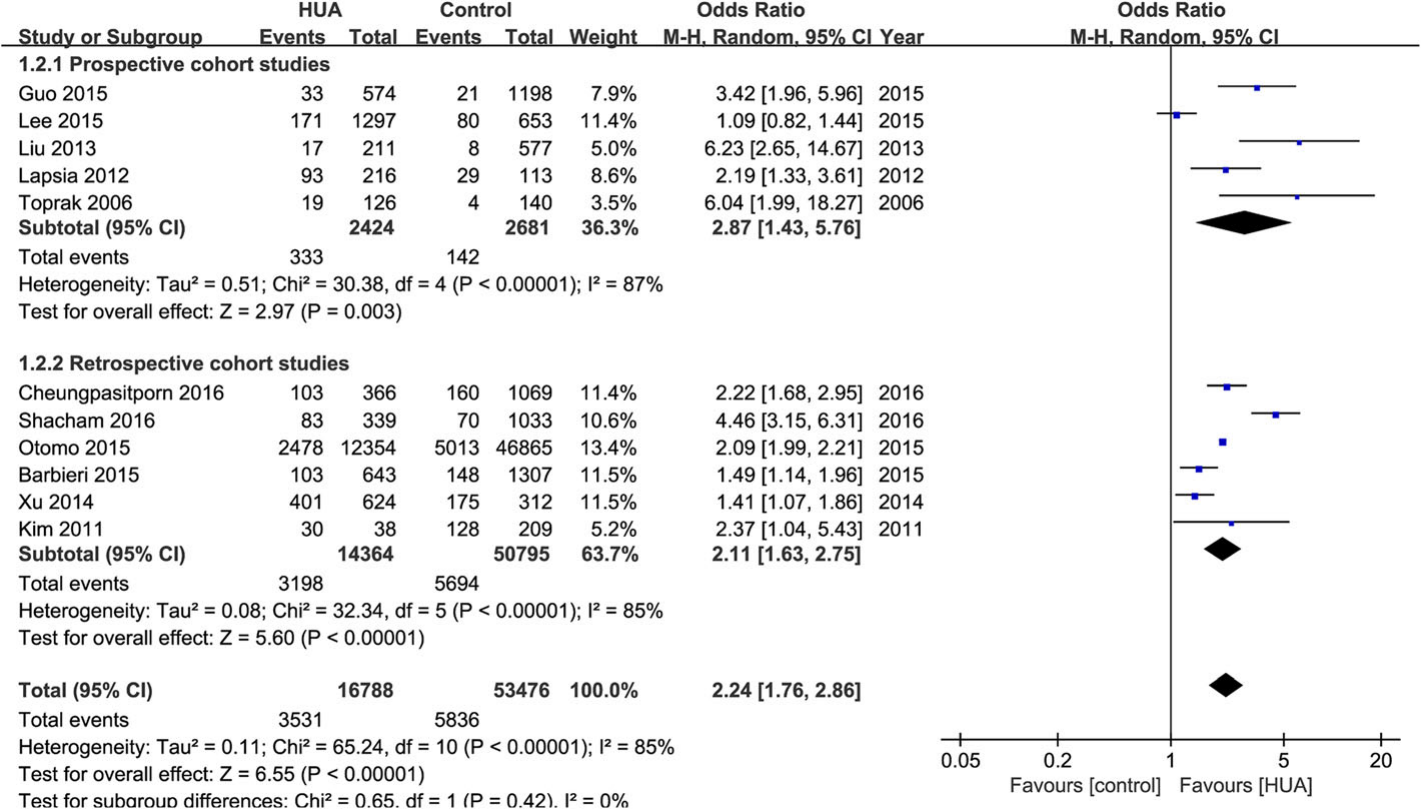
Effects of hyperuricemia on incidence of acute kidney injury in prospective and retrospective studies

### Subgroup analysis

Although the pooled rates of AKI incidence after cardiac surgery in hyperuricemia and control group were 34.3% (95% CI 4.4-85.5%) and 29.7% (95% CI 4.6-78.7%) respectively (OR 1.24, 95% CI 1.96-1.60, *p* = 0.10), the AKI incidence after percutaneous coronary intervention (PCI) were 16.0% (95% CI 8.6-27.7%) and 5.3% (95% CI 2.5-10.9%) respectively (OR 3.24, 95% CI 1.93-5.45, *p* < 0.00001) (Figs. 2b and 5).

**Fig. 7:**
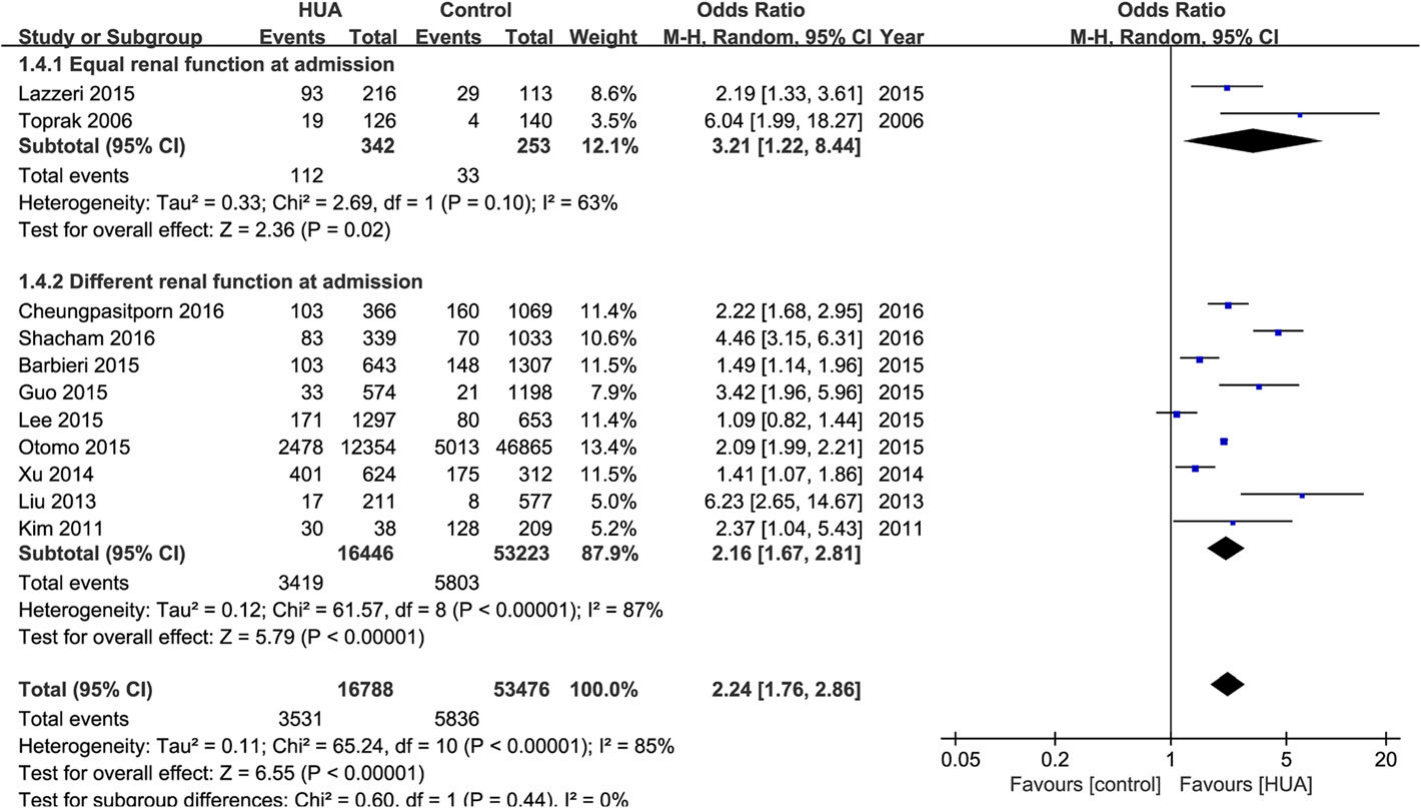
Effects of hyperuricemia on incidence of acute kidney injury in patients with or without equal renal function at admission

We also conducted subgroup analysis of prospective and retrospective cohort studies (Fig. 6). The pooled ORs of hyperuricemia on AKI were 2.87 (95% CI 1.43-5.76) and 2.11 (95% CI 1.63-2.75) respectively. In addition, to reduce the bias of included patients, we also analyzed studies with or without equal renal function, which was defined as serum creatintine or estimated glomerular filtration rate (eGFR) without significant different at admission between hyperuricemia and control groups. There were significant differences in renal function at admission between hyperuricemia and control groups in most of the included studies, while only two studies with equal renal function were included, and the pooled OR was 3.21 (95% CI 1.22-8.44, *p* = 0.02) (Fig. 7).

### Effects of SUA on hospital mortality

**Fig. 8:**
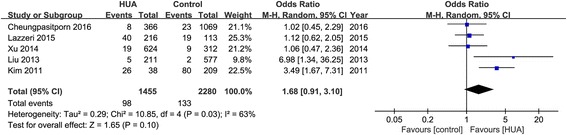
Effects of hyperuricemia on hospital mortality

Five studies with 3735 patients provided the hospital mortality. The pooled rates of hospital mortality in hyperuricemia group and control group were 8.9% (95% CI, 2.1-30.8%) and 5.0% (95% CI, 1.0-21.9%) respectively (OR 1.68, 95% CI 0.91-3.1, *p* = 0.083) (Figs. 2c and 8). The relationship between hyperuricemia and hospital mortality was not significant.

### SUA levels in AKI and Non-AKI groups

Five studies assessed the SUA levels in AKI and Non-AKI groups. The pooled pre-operative SUA levels were higher in AKI group (376.35 μmol/L, 95% CI 321.76-430.93 μmol/L) than in Non-AKI group (317.09 μmol/L, 95% CI 304.50-329.68 μmol/L) (Std diff in means 0.860, 95% CI 0.334-0.112, *p* = 0.010) (Fig. 2d).

### Publication bias

The funnel plots showed no evidence of publication bias. Egger’s test for a regression intercept gave a *p*-value of 0.696 for effects of hyperuricemia on incidence of AKI, indicating no publication bias.

## Discussion

AKI is one of the most serious complications with a reported mortality rate of 15% in hospitalized patients [15]. Our meta-analysis showed that HUA is a critical and potential risk factor for the incidence of AKI, not only in preoperative patients as reported previously but also in all hospitalized patients.

In this meta-analysis, we found that the pooled rates of AKI incidence in hyperuricemia group were much higher than that in the control group. The underlying reasons were analyzed as follows. Firstly,majority of uric acid is excreted by the kidneys and accounts for 70%. It should be noted that approximately 90–95% of the filtered uric acid from glomerular is absorbed, mostly by proximal tubules [16, 17]. Secreted uric acid by the renal tubules is very little. Consequently the SUA concentration depends on glomerular filtration and subsequent tubular reabsorption function. There is mounting evidence to consider SUA as a clear marker for chronic kidney disease or an independent risk factor for the development of chronic kidney disease [18, 19]. A number of studies demonstrated that pre-existing chronic kidney disease increases the risk of AKI. Ishani et al. reported that the incidence of AKI was 8.8% in patients with chronic kidney disease versus 2.3% in patients without chronic kidney disease [20]. Pannu N et al. found that the risk of AKI was 18-fold higher in patients with an eGFR less than 30 ml/min/1.73 m^2^ than in those with an eGFR more than 60 ml/min/1.73 m^2^ [21]. Therefore, patients with increased SUA may already have the subclinical chronic renal dysfunction, leading them to be more vulnerable to AKI. In addition, we did an adjustment for the important covariate baseline GFR or serum creatinine. Unfortunately, there were only two included studies with equal renal function at admission, the results from which was more convincing.

Seconding, an elevated SUA concentration has been found to be associated with damage of impartment organs and result to many diseases such as hypertension [17, 22], metabolic syndrome [23], atherosclerosis [24], myocardial infarction [25], diabetes mellitus [4], stroke [26] and so on. All of the above diseases are most common risk factor of AKI, which make it sense that the incidence of AKI in the hyperuricemic patients is higher than those in the normouricemic patients.

A number of studies supported that uric acid is an independent risk factor of cardiovascular disease. The incidence rate of cardiovascular disease in patients with hyperuricemia is higher than that in the normal population [27]. A meta-analysis showed that incidence of coronary heart disease (CHD) in the hyperuricemic patients was 1.34 times (95% CI 1.19-1.49) than that in the normouricemic patients [5]. Patients with CHD combined with hyperuricemia have higher incidence of myocardial infarction. The global number of cardiac surgeries or PCI each year is approximately 2 million [28, 29] and one of the most common and serious post-operative complications is AKI. A current meta analysis found that the incidence of AKI after cardiac surgery was 22.3% around the world (95% CI 19.8-25.1) [2]. The incidence of PCI-induced AKI has been estimated between 2% and 30% depending mainly on baseline renal function, which is increasing along with the higher prevalence of CHD year by year [15, 29]. Our results suggest that higher pre-PCI SUA increased risk of AKI. We speculated that the patients with increased SUA maybe undergo more PCI, consequently have more incidence of AKI. In addition, it was found contrast agents have a uricosuric effect through enhancing renal tubular secretion of uric acid [30], which may promote renal injury caused by possible nephrotoxic effect of uric acid. However, there are more complex risk factors and mechanisms of AKI incidence after cardiac surgery than PCI, which led to less difference of the pooled rate of AKI between hyperuricemia and control group. Moreover, there need more studies to confirm the prognostic role of SUA in AKI incidence after cardiac surgery.

Finally, it is well-known that AKI is resulted from multiple and interactive pathways. Uric acid itself can cause AKI due to several mechanisms ranging from direct tubular toxicity (crystal induced injury) [9] to indirect injury (secondary to vasoconstriction, oxidative stress, inflammatory and so on). In both animal and human models, uric acid is found to inhibit proliferation and migration of endothelial cell and cause dysfunction and apoptosis of endothelial cell [31, 32]. Animal experimental studies suggest that uric acid may cause renal vasoconstriction via inhibiting of renal nitric oxide synthase to reduce product of nitric oxide in endothelial cell [31] and via stimulating of the renin-angiotensin system [32]. Renal vasoconstriction is a common pathogenic factor in the progression of AKI [33]. Inflammatory and oxidative stress are two of important mechanisms of AKI [34]. Experimentally, it has been found that uric acid activates inflammatory transcription factor nuclear factor-κB signaling pathway [35]. Increasing SUA also stimulates the expression of pro-inflammatory systemic cytokine i.e. tumor necrosis factor α [36], and the local chemokines, i.e. monocyte chemotactic protein 1 in the kidney [37]. High SUA levels induced oxidative damage of proximal tubule cell by activating nicotinamide adenine dinucleotide phosphate (NADPH) oxidase [38]. Therefore, SUA may be involved in the progress of AKI and contribute to higher incidence of AKI in the patients with hyperuricemia. Regardless of whether elevated SUA is solely a predictive factor of AKI or an independent risk factor of AKI, careful attention is warranted.

Thus, we wonder if uric acid lowering therapy could decrease the risk for developing AKI. At present, no trials showed that lowering SUA may provide benefit in preventing AKI. Allopurinol was once used in the hyperuricemic patients before cardiovascular surgery to reduce oxidative stress and then improve cardiovascular outcomes [39]. However, it was found that allopurinol couldn’t prevent the incidence of AKI after cardiac surgery in these studies [40]. After that, researchers confirmed the protective role of allopurinol in the renal ischemia/reperfusion injury in rats [41, 42]. In addition, in the cisplatin-induced AKI models, the uric acid lowering drugs rasburicase [43] and febuxostat [44] could attenuate renal injury by their antioxidant, anti-inflammatory, and cytoprotective effects. A prospective, randomized pilot trial with 26 cardiac surgery patients with hyperuricemia showed that there was no significant difference of postoperative serum creatinine between subjects receiving rasburicase and the control group. However, urine NGAL tended to be lower in the rasburicase group, which suggested that lowing uric acid before surgery might protect against renal tubular injury [45]. In Sezai A et al. study, febuxostat had a renoprotective effect with a significant earlier decrease of UA after cardiac surgery in hyperuricemic patients compared with allopurinol [46]. Therefore, we postulated that early intervention to decrease SUA levels may lower the risk of developing AKI.

### Strengths and limitations

To the best of our knowledge, this study is the first to systematically evaluate the indicated effect of SUA on the incidence of AKI especially after cardiac surgery and PCI. It included data more than 75,000 patients from 18 studies. We analyzed these studies in detail considering the effect of renal function at admission and study design.

However, the present study may have limitations. Firstly, if there were more randomized controlled trials with high quality and large samples in this meta-analysis, these results would be more convincing. Secondly, Kanda et al. indicated that SUA level has a U-shaped association with loss of kidney function and low SUA (male <5 mg/dl; female <3.6 mg/dl) is also a candidate predictor of chronic kidney disease [47]. We are only focused on the role of hyperuricemia in AKI without referring hypouricemia which will need more studies in the future.

## Conclusion

This meta-analysis demonstrated that elevated SUA levels could be associated with an increased risk of developing AKI especially in the patients after cardiac surgery and PCI.

## Abbreviations

AKI: Acute kidney injury
CHD: Coronary heart disease
GFR: Glomerular filtration rate
OR: Odds ratio
PCI: Percutaneous coronary intervention
SUA: Serum uric acid
